# Applications of Organic and Inorganic Amendments Induce Changes in the Mobility of Mercury and Macro- and Micronutrients of Soils

**DOI:** 10.1155/2014/407049

**Published:** 2014-10-23

**Authors:** Mercedes García-Sánchez, Adéla Šípková, Jiřina Száková, Lukáš Kaplan, Pavla Ochecová, Pavel Tlustoš

**Affiliations:** Department of Agro-Environmental Chemistry and Plant Nutrition, Faculty of Agrobiology, Food and Natural Resources, Czech University of Life Sciences, Suchdol, 16521 Prague 6, Czech Republic

## Abstract

Both soil organic matter and sulfur (S) can reduce or even suppress mercury (Hg) mobility and bioavailability in soil. A batch incubation experiment was conducted with a Chernozem and a Luvisol artificially contaminated by 440 mg*·*kg^−1^ Hg showing wide differences in their physicochemical properties and available nutrients. The individual treatments were (i) digestate from the anaerobic fermentation of biowaste; (ii) fly ash from wood chip combustion; and (iii) ammonium sulfate, and every treatment was added with the same amount of S. The mobile Hg portion in Chernozem was highly reduced by adding digestate, even after 1 day of incubation, compared to control. Meanwhile, the outcome of these treatments was a decrease of mobile Hg forms as a function of incubation time whereas the contents of magnesium (Mg), potassium (K), iron (Fe), manganese (Mn), copper (Cu), zinc (Zn), and phosphorus (P) were stimulated by the addition of digestate in both soils. The available calcium (Ca) contents were not affected by the digestate addition. The experiment proved digestate application as the efficient measure for fast reduction of mobile Hg at extremely contaminated soils. Moreover, the decrease of the mobile mercury portion was followed by improvement of the nutrient status of the soils.

## 1. Introduction

Industrial activities have increased the proportion of Hg in the atmosphere and oceans and have contaminated a number of local environments [1]. From the ecotoxicological point of view, critical limits of Hg (given as soil element contents above which unacceptable effects are expected) are substantially lower than values derived for other metals such as Cd, Cu, Ni, Pb, and Zn [2]. Li et al. [3] compared the mobility and plant-availability of risk elements from industrially contaminated soil where the soil-to-plant transfer coefficients were in the order of Cd > Zn > Cu > Hg > As > Pb, confirming the relatively low availability of soil Hg for various vegetables. Rodrigues et al. [4] observed the water-soluble contents of Hg in highly contaminated sediment and soil samples (total Hg contents even higher than 3000 mg*·*kg^−1^) to be less than 1.2% of the total Hg content. Boszke et al. [5] classified the divalent and elemental Hg bounds to humic matter/organic matter as the “semimobile” element portion and observed low portions of the water-soluble Hg species as well.

Luo et al. [6] suggested that soil organic matter and nitrogen were the important sinks for Hg in the soils. The good capacity of Hg for adsorption and complexation in the solid media resulted in limited bioaccessibility of this element, which was reported by Hassen et al. [7]. Distribution coefficients for Hg^2+^ binding by humic acids were determined by Khwaja et al. [8], confirming that the calculated concentration of free Hg^2+^ at equilibrium is very low. Also, Heeraman et al. [9] observed decreasing Hg mobility and plant-availability in the organic matter-treated soil. The importance of soil organic matter for Hg mobility and bioavailability in soil samples is known and well described [5, 10]. As observed by Yao et al. [11], the addition of humus can either suppress or promote Hg bioavailability depending on the soil composition. In this context, the effect of a particular humus fraction on Hg bioavailability is related to its ability to convert Hg bound by solid phases into soluble complexes, as well as the stability of the released complexes. On the contrary, the presence of dissolved organic matter (DOM) can significantly reduce maximum Hg adsorption capacity and even promote Hg desorption from the soils [12].

Zagury et al. [13] evaluated the potential mobility and plant-availability of Hg in the highly contaminated soils by chlor-alkali plants, where the total Hg contents in soil reached up to 11500 mg*·*kg^−1^. Although the water extractable Hg portion was relatively low with regard to the high total content, it represented significant concentrations correlating with Hg uptake by experimental plants.

Reis et al. [14] observed that the presence of Hg in the mobile phase could be related to Mn and aluminum (Al) soil contents. A positive relation between Hg in the semimobile fraction and the Al content was also observed. On the contrary, organic matter and S contents contributed to Hg retention in the soil matrix, reducing the mobility of the metal. Sulfide minerals are known to be effective adsorbents for Hg(II) being the primary sink for Hg in the environment [15]. In this context, Hesterberg et al. [16] demonstrated the preferential binding of Hg(II) to reduced organic S sites. Subsequently, similar observations were provided and described in soils as mentioned by Remy et al. [17]. Concentration of MeHg is negatively correlated with soil total organic matter and total S and is influenced by the soil total Hg concentration [17]. Åkerblom et al. [18] highlighted that long-term chronic SO_4_^2−^ deposition at rates similar to those found in polluted areas of Europe and North America increase the capacity of peatlands to methylate Hg and store MeHg. Competitive relationships between Hg and other metals in soil were observed by Jing et al. [19], where desorption of adsorbed Hg increased with elevated concentrations of added Cu or Zn.

In our investigation, a laboratory batch incubation experiment was conducted with Chernozem and Luvisol differing in their physicochemical parameters and the available nutrient contents. Digestate, the bio-waste originating from biogas production plants, was applied as a S-rich source of organic matter. Alternatively, wood ash from biomass combustion plants was applied as a different source of S and other macro- and micronutrients. As proven by Ochecová et al. [20], the plant-availability of the risk elements in the contaminated soil decreased after ash application, whereas the nutrient contents tended to increase. To separate the effect of organic matter and S in the soil, inorganic source of S, ammonium sulfate, (NH_4_)_2_SO_4_, was applied, as well. The main objectives of the study were as follows: (i) to assess the ability of the individual treatments to immobilize Hg in the artificially contaminated soil and (ii) to evaluate the potential interactions between Hg sorption in the experimental soil and the mobility of the essential macro- and microelements in these soils.

## 2. Materials and Methods

### 2.1. Soils and Ameliorative Materials

The following two soils, differing in their physicochemical characteristics, were selected for the experiment: (i) uncontaminated Chernozem with a cation exchange capacity (CEC) of 230 mmol kg^−1^, a pH level of 7.5, and an oxidizable carbon content (Cox) of 2.6%, and (ii) uncontaminated Luvisol with a CEC of 145 mmol kg^−1^, a pH level of 6.5, and a Cox of 1.7%. Nutrient contents and other characteristics in both soil samples are summarized in Table 1. Soils were sampled from a depth of 20 cm, immediately after which they were homogenized, sieved through a 5 mm diameter mesh, and kept at room temperature. For the experimental incubation soils, samples were sieved again using a 2 mm mesh and kept at 4°C until use. The fly ash (pH 12.1) was produced by the combustion of wood ash produced in two reactors (1.8 MW and 0.6 MW). The digestate sample (pH 8.2) originated from a biogas station (1732 kW/h), where the digested material consisted of sugar beet pulp (50%), marc of fruit (42%), and silage maize (8%). The macro- and micronutrient contents in both ameliorative materials are summarized in Table 2. As an inorganic amendment, solid particles of (NH_4_)_2_SO_4_ were used (Reagent from Fisher Scientific).

### 2.2. Experimental Design

For the experimental incubation soils, 100 g of Chernozem and Luvisol soils were placed into polypropylene bottles and immediately after were brought to 60% moisture saturation. Then, half of the soil samples were artificially contaminated with Hg by adding 60 mg of HgCl_2_ to reach a concentration of 440 mg*·*kg^−1^ of Hg. Subsequently, organic and inorganic amendments: (1) ash, (2) digestate, and (3) (NH_4_)_2_SO_4_, were applied both to contaminated and noncontaminated soils. The rate of amendment was calculated for 600 mg S per kg of soil as follows: (1) ash: 1.5 g, (2) digestate: 10 g, and (3) NH_4_SO_4_: 0.25 g per bottle.

Soils that were contaminated and noncontaminated with Hg were thoroughly mixed and incubated at 28°C for 21 days. To evaluate the mobility of Hg in both soils and interactions with macro- and micronutrients as well, soil samples were collected after 1, 2, 3, 4, 7, 14, and 21 days of incubation. Three replicates were set up per treatment.

### 2.3. Extraction of Soluble Portions of Hg and Macro- and Micronutrients

For the determination of bioavailable element portions in soils during the experiment, 0.5 g of each sample was added to 10 mL of 0.11 mol L^−1^ solution of CH_3_COOH and shaken overnight [21]. Each extraction was carried out in three replicates. For the centrifugation of extracts, a Hettich Universal 30 RF (Germany) instrument was used. The reaction mixture was centrifuged at 3000 rpm (i.e., 460 g) for 10 minutes at the end of each extraction procedure, and the supernatants were kept at 6°C prior to measurements. Prior to the analysis, extracts were acidified with a mixture of acids (HNO_3_ : HCl = 3 : 1). For the determination of nutrient status in the experimental soils before the experiment, the Mehlich III extraction procedure (0.2 mol L^−1^ of CH_3_COOH + 0.25 mol L^−1^ of NH_4_NO_3_ + 0.013 mol L^−1^ of HNO_3_ + 0.015 mol L^−1^ of NH_4_F + 0.001 mol L^−1^ of EDTA) at the ratio of 1 g of soil per 10 mL of the extraction mixture for 10 min [22] was applied.

### 2.4. Determination of Hg

Hg content in the extracts was measured by inductively coupled plasma mass spectrometry (ICP-MS, Agilent 7700x, Agilent Technologies Inc., USA). The auto-sampler ASX-500, a three-channel peristaltic pump, and MicroMist nebulizer equipped the ICP-MS. Calibration solutions were prepared in diluted single element ICP-MS standards as 0.1–100 *μ*g L^−1^ for Hg and the isotope Hg(202) was measured. As an internal standard, Pt(195) was used at the concentration of 100 *μ*g L^−1^.

### 2.5. Determination of Macro- and Micronutrients

Inductively coupled plasma-atomic emission spectrometry (ICP-OES, Agilent 720, Agilent Technologies Inc., USA) equipped with a two-channel peristaltic pump, a Struman-Masters spray chamber, and a V-groove pneumatic nebulizer made of inert material was applied for the determination of Cu, Fe, Mn, Zn, P, and S in the extracts (the experimental conditions were as follows: power of 1.2 kW, plasma flow of 15.0 L*·*min^−1^, auxiliary flow of 0.75 L*·*min^−1^, nebulizer flow of 0.9 L*·*min^−1^), whereas flame atomic absorption spectrometry (F-AAS, Varian 280FS, Varian, Australia; air flow of 13.5 L*·*min^−1^, acetylene flow of 2.2 L*·*min^−1^, burner height of 13.5 mL, nebulizer uptake rate of 5 mL*·*min^−1^) was used for Ca, Mg, and K determination in the extracts.

### 2.6. Determination of Total Nutrient Contents in the Ameliorative Materials

For determination of total element contents in the ash, nondestructive X-ray fluorescence (XRF) spectrometry (Spectro IQ, Kleve, Germany) was used; the target material was palladium and the target angle from the central ray was 90°. The focal point was a 1 mm × 1 mm square, and the maximum anode dissipation was 50 W with 10 cfm forced-air cooling. The instrument was equipped with the Barkla crystal HOPG. The tested samples were pressed into pellets; this involved mixing 4.0 g of ash (particle size 15–20 *μ*m) with 0.9 g of the binding additive (HWC Hoechst wax, Germany) for 10 min with a pressing power of 80 kN. The determination was performed in the Institute of Rock Structure and Mechanics, Academy of Sciences of the Czech Republic.

The digestate sample was decomposed by pressurized wet ashing as follows: aliquots (~0.5 g) of air-dried samples were decomposed in a digestion vessel with 10 mL of* Aqua regia* (i.e., nitric and hydrochloric acid mixture in the ratio 1 : 3). The mixture was heated in an Ethos 1 (MLS, Germany) microwave assisted wet digestion system for 33 min at 210°C. ICP-OES and F-AAS were then applied as described in the previous subchapter.

### 2.7. Statistics

The data obtained were subjected to Dixon's test for the identification of outliers (significance level *α* = 0.05) using Microsoft Office Excel 2007 (Microsoft Corporation, USA). Subsequently, one-way analysis of variance was used at the significance level *α* = 0.05 using the Statistica 12 program (StatSoft, USA).

## 3. Results and Discussion

### 3.1. Changes in Hg Mobility in the Treated Soils

**Figure 1:**
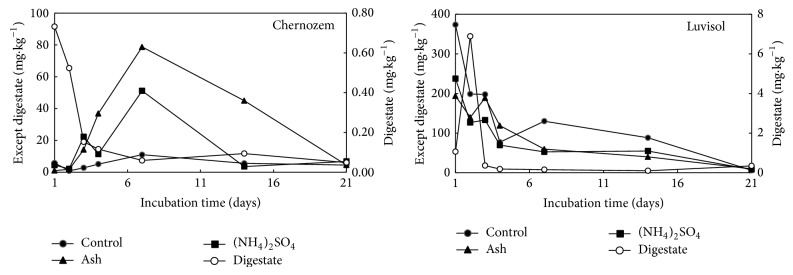
The concentrations of Hg extractable with 0.11 mol L^−1^ acetic acid within the incubation experiment (mg*·*kg^−1^) according to the individual treatments.

The mobile Hg contents affected by the individual treatments and their variability during the incubation experiment are summarized in Figure 1. In the treatments without artificial Hg application, the mobile Hg contents were under the detection limit of ICP-MS. Similarly to our previous observations [23], Ruggiero et al. [24] also documented that most of the Hg in the long-term polluted soils was scarcely mobile and available. The Hg contents in digestates and fly ash are usually low as well [25, 26] and did not affect the mobile portions of Hg in our experiment. The extractable Hg contents differed according to the physicochemical parameters of the used soils and to the individual treatments. In Chernozem, the extractable Hg contents were low regardless of the treatment at the beginning and end of incubation. Within the 3rd and 7th day of incubation, the mobile Hg portions increased in all treatments (including control) except for the digestate. Similar course of Hg mobility changes were observed by Bower et al. [27] in the experiments studying the mercury adsorption onto pyrite indicating the formation of nonmobile sulfides over time. In the Luvisol, the mobile Hg portions decreased during the incubation, whereas they dropped to the levels reached in Chernozem by the end of the experiment. As stated by Müller et al. [28] soil Hg contamination can cause reduced microbial biomass at the contaminated sites. However, some microorganisms have developed mechanisms to adapt to Hg, that is, Hg-resistant bacteria. Thus, the changes in Hg mobility observed throughout the experiment could be partially attributed to different communities of soil microorganism present in both Chernozem and Luvisol.

Therefore, among the individual treatments, digestate was shown to be the most effective Hg immobilizing agent, whereas fly ash seemed to be less effective, and no significant difference was reported comparing the ammonium sulfate treatment and untreated control except the faster increase of mobile Hg content in 3rd and 4th days of the incubation indicating potential role of increased portion of mobile sulfur as mentioned below. The effectiveness of individual treatments as well as the temporal changes in mobile Hg portions were also affected predominantly by the soil where higher sorption capacity and organic matter content in Chernozem resulted in lower mobile portions of Hg in all the treatments except increased mobility of Hg after ash application in 7th and 14th day of the incubation. These results also indicated that S content in the ameliorative materials was not the main factor controlling the Hg mobility in the soils. Luo et al. [6] reported a low relationship between S and Hg contents in soils with low total organic carbon (~2%), as in our case, where the carbon content was not increased by the addition of ammonium sulfate and ash. In the opposite, the Hg behaviour in soils strongly differed if digestate with high content of both S and total carbon content was applied.

Higher organic carbon content in the soil can enhance both soil microbial activities and the retention of total Hg and MeHg in soil [29]. Soil microorganisms need essential metals for their metabolism, which are often required in low concentrations and act as enzyme cofactors [30]. Therefore, high contents of macro- and micronutrients in both ash and digestate (Table 2) can be beneficial for the enhancement of the microbial activity in soils. Limited Hg mobility* via* complexation with soil organic matter was already described [9]. Ravichandran [31] reviewed the formation of extremely strong ionic bonds between Hg and reduced S sites in soil organic matter supporting the importance of the mutual role of S and organic matter in Hg immobilization in soil. Therefore, the Hg desorption increased with elevating concentrations of dissolved organic matter [10]. In our case, the dissolved organic matter after 1 day of incubation varied between 71.9 mg*·*kg^−1^ (control) and 1070 mg*·*kg^−1^ (digestate) in Chernozem and between 21.2 mg*·*kg^−1^ (control) and 56.4 mg*·*kg^−1^ (digestate) in Luvisol. After 7 days of incubation, the DOM contents increased even 22-fold in the digestate-treated Luvisol, whereas the maximum 1.5-fold increase was observed in Chernozem. Therefore, our results indicate that more complex factors can change the Hg mobility in soil than solely the content and solubility of organic carbon in soil. For example, the affinity of Hg to bind to metal oxides should be taken into account [32]. Also, the role of some soil bacteria which are able to degrade Hg compounds into metallic Hg by the action of specific enzymes encoded by the* mer* genes and then be released into the surrounding environment should be considered [33]. Thus, the decrease of mobile Hg in soil could be partially figured in the volatilization of this element during incubation. This assumption remains to be verified in further research. In our investigation, the experiments were concerned on the description of potential decrease of mobile Hg content without exact resolution between immobilization/volatilization ratios after the individual treatments.

### 3.2. The Effect of Hg and/or Ameliorative Materials on Mobile Contents of Macro- and Micronutrients in the Soil

The mobile macro- and microelement contents affected by the individual treatments and/or duration of incubation are summarized in Tables 3, 4, 5, 6, 7, 8, 9, 10, and 11. The presence of digestate showed a predominant effect on the mobile portions of most of the elements among all the treatments. The mobile element contents significantly increased after digestate application for most of the determined nutrients, except Ca and Cu in Chernozem (because of its low availability in the ameliorative materials). More apparent increase of mobile element contents after application of digestate was observed for Luvisol compared to Chernozem due to higher sorption capacity of Chernozem in accordance with their higher CEC level. For example, Table 4 shows 5-fold increase of mobile Mg contents in the digestate treated Luvisol compared to up to 40% increase of mobile Mg in Chernozem.

Möller and Müller [34] reviewed recent research about nutrient availability after the field application of digestate and stated that there is no available information concerning the availability of S, although digestate seems to be a good source of S in soil; this was also observed in our case (Table 2) where the S content in the digestate sample reached up to 0.6%. Similarly, they stated that there were many published studies describing the effect of anaerobic digestion on micronutrient distribution and bioavailability in sewage sludge, but rarely any concerning digestates. Moreover, the availability of micronutrients in the digestate can be affected by the wide complex of various factors such as precipitation as sulfide, carbonate, phosphates, and hydroxides, sorption to the solid fraction, either biomass or inert suspended matter, and the formation of complexes in solution with intermediates and compounds produced during anaerobic digestion [34]. The application of digestate results in an improvement of crop yields compared to inorganic fertilizer. Moreover, analysis of soil solution showed that there was less potential for the loss of nutrients* via* leaching [35] in the digestate treated soil. Also, Frøseth et al. [36] observed that the field application of digestate contributed to higher soil aggregate stability. According to Fernández-Delgado Juarez et al. [37], amending soils with digestate resulted in a higher nutrient content as well as more efficient soil microbial community relative to the variants treated with farmyard manure. Therefore, the application of digestate seemed to be the effective measure for immobilization of Hg in soil together with increase of mobile nutrients in these soils.

The application of wood fly ash as a potential source of available nutrients in the soil is widely discussed in the literature [38, 39]. The benefits on the growth of the plants as the result of an increase in available P, Ca, Mg, K, and B and a decrease in Al toxicity was described [38]. Steenari et al. [40] tested the release of macro- and microelements from various ash samples, whereas low leaching rates were observed for the important plant nutrients P and Mg, as well as for Fe, Mn, Cu, and Zn, up to 50% of total K was released during the batch leaching test. In our case, the extractable element contents in the ash amended samples differed according to the experimental soil, where Ca, Fe, and Cu levels remained unchanged compared to the control. On the contrary, the extremely high Zn level in the ash (Table 2) resulted in the significant increase in the extractable Zn portion of the ash-treated soil regardless of the soil type (Table 11). A similar pattern was reported for Mg, where the increase in the extractable Mg portion was more apparent in Luvisol (Table 4). Whereas in the Luvisol the mobile Mg contents increased twice after ash application, the mobile portion of Mg in Chernozem rised only by 10–15%. The low mobility of micronutrients in various ash samples was also confirmed by Száková et al. [41]. The K, Mn, and P extractable levels tended to increase compared to controls (significance of the differences at *P* < 0.05 was unambiguously proved only in the case of Mn, see Table 8) but were significantly lower compared to digestate application, not confirming the high K leachability from ash samples observed by Steenari et al. [40]. Although the total S contents added* via* the individual treatments were comparable, the mobile portions of ash-derived S were lower compared to those after the application of digestate and ammonium sulfate. Ochecová et al. [20] observed increasing mobile portions of Ca, P, K, Mg, and Mn in the fly ash-treated soil after a 3-year model pot experiment. However, the effects were significant for the 3–6 fold higher ash rate compared to our experiment. Thus, the increase of mobile nutrient contents in soils will manifest at higher ash rates compared to our experiment.

The interrelationships between soil Hg and other soil element contents described by Reis et al. [14] indicate that the presence of Hg in the mobile phase could be related to Mn and Al soil contents. Furthermore, an antagonistic effect of Mn against Hg is suggested. Our data tended to increase of mobile Mn contents during the incubation (Table 8) as related to decreasing mobile Hg (Figure 1). Similarly, Sierra et al. [42] observed negative significant correlation between the available Mn in the rhizosphere and Hg content in plants. On the contrary, S content contributed to Hg retention in the soil matrix, reducing the mobility of the metal [14]. In our case, the changes of Hg mobility in soils (Figure 1) did not reflect the changes in mobile portion of S during the incubation experiment (Table 10). In contrast, the presence of sulfates seems to favor Hg uptake by the plant. There was a positive significant correlation between the sulfate concentration in the rhizosphere and the Hg within the aerial and root parts of plants [42]. However, only total S extracted with the 0.11 mol L^−1^ solution of CH_3_COOH was determined by the ICP-OES and a portion of mobile sulfates in the extract was not determined in our case and requires further research. The competition between Hg and Cu and Hg and Zn in soils described by Jing et al. [19] was not confirmed in our case. For Fe, Mehrotra and Sedlak [43] and Rhoton and Bennett [44] highlighted Hg immobilization* via* sorption and/or the complexation of Hg with Fe compounds in soil. This statement seems to be confirmed for Chernozem, whereas the opposite pattern was observed in Luvisol. In Chernozem, the mobile portions of Fe decreased significantly after digestate application on the Hg amended samples compared to the unamended ones. In Luvisol, the mobile Fe contents increased in the Hg + digestate amended samples since 2nd day of incubation with maximum at 7th day suggesting competitive relationships of Fe and Hg in this case. The complexity of Hg sorption on Fe/Mn oxides was documented by Liang et al. [45], where the role of amorphous/crystalline Fe and Mn hydroxides, humic acids content, and also chlorine concentrations were mentioned. Šípková et al. [46] observed a negative correlation between Hg content bound to the humic acids and the content of Mg, Mn, and Fe. Therefore, the more detailed information concerning soil components, humic acid portions in the digestate, as well as the importance of the application of HgCl_2 _ compared to the other Hg compounds remains to be elucidated in further research.

## 4. Conclusions

Although the response of Hg contaminated soils in different ameliorative materials was affected by the individual parameters of the soils, especially by the different soil sorption capacity and organic matter contents in these soils, digestate proved to be the most effective for the immobilization of Hg in soil. Contrary to the other S-bearing measures such as wood fly ash and ammonium sulfate, in the case of digestate, the Hg immobilizing effectiveness resulted from the cooperation of various factors such as S and organic matter content. Moreover, the digestate application can result in an improvement in the macro- and micronutrient status of the soil, where mobile and theoretically plant-available portions of these elements increased in particular. Thus, the field application of organic matter-rich biowaste such as digestate seems to be reasonable for the disposal of this type of material, leading to a decreased environmental risk of Hg contamination in soil.

## Figures and Tables

**Table 1:** Main physicochemical characteristics of the experimental soils.

Soil type	Luvisol	Chernozem
NRSC Soil Texture	Silt loam	Silt loam
Clay (<0.002 mm) [%]	5.38	2.18
Silt (0.002–0.05 mm) [%]	68.14	71.80
Sand (0.05–2 mm) [%]	26.48	26.03
Location	50°4′22′′N, 14°10′19′′E	50°7′40′′N, 14°22′33′′E
Altitude (m a.s.l.)	410	286
P Mehlich III∗ (mg kg^−1^)	100	91
K Mehlich III∗ (mg kg^−1^)	80	230
Mg Mehlich III∗ (mg kg^−1^)	110	240
Ca Mehlich III∗ (mg kg^−1^)	3600	9000

^*^Šípková et al. [23].

**Table 2:** Nutrient contents in the dry matter of ameliorative materials.

Element	Fly-ash	Digestate
P (%)	1.29 ± 0.01	1.20 ± 0.01
K (%)	7.74 ± 0.02	2.12 ± 0.01
Mg (%)	1.44 ± 0.02	0.49 ± 0.02
Ca (%)	13.4 ± 0.1	3.15 ± 0.01
S (%)	4.07 ± 0.01	0.60 ± 0.01
Cu (%)	0.020 ± 0.001	0.004 ± 0.001
Fe (%)	2.79 ± 0.01	0.18 ± 0.01
Mn (%)	1.29 ± 0.01	0.02 ± 0.00
Zn (%)	3.58 ± 0.08	0.03 ± 0.00

**Table 3:** The concentrations of Ca extractable with 0.11 mol L^−1^ acetic acid within the incubation experiment (mg*·*kg^−1^); the averages marked by the same letter did not significantly differ at *P* < 0.05 within individual columns; data are presented as mean ± standard deviation (*n* = 3).

Chernozem	Day 1	Day 2	Day 3	Day 4	Day 7	Day 14	Day 21
Control	7555 ± 331^a^	7655 ± 183^a^	8390 ± a213^a^	9674 ± 219^a^	9034 ± 238^ab^	9856 ± 1191^ab^	6040 ± 270^a^
Control + Hg	8074 ± 260^a^	7694 ± 359^a^	7467 ± a677^a^	9607 ± 193^a^	8891 ± 702^a^	8951 ± 236^ab^	7865 ± 983^abc^
Digestate	8282 ± 735^a^	8094 ± 155^a^	8049 ± a687^a^	10122 ± 206^a^	8886 ± 362^a^	11004 ± 926^b^	12402 ± 2330^bc^
Digestate + Hg	8108 ± 232^a^	9656 ± 282^a^	7824 ± a454^a^	11360 ± 1521^a^	10104 ± 205^b^	10232 ± 499^ab^	11286 ± 1243^c^
Ash	8448 ± 548^a^	8246 ± a282^a^	8746 ± a272^a^	9898 ± 213^a^	9661 ± 466^ab^	9633 ± 203^ab^	9835 ± 2134^abc^
Ash + Hg	8164 ± 921^a^	8072 ± a511^a^	8717 ± a611^a^	10910 ± 898^a^	9862 ± 300^ab^	10599 ± 387^ab^	9359 ± 1118^abc^
(NH_4_)_2_SO_4_	7330 ± 57^a^	7409 ± a201^a^	8037 ± a311^a^	10134 ± 1290^a^	8942 ± 307^a^	8893 ± 990^a^	10045 ± 2115^abc^
(NH_4_)_2_SO_4_ + Hg	8041 ± 796^a^	7393 ± a359^a^	8350 ± 1151^a^	9820 ± 207^a^	8991 ± 382^ab^	10238 ± 790^ab^	7031 ± 3008^abc^

Luvisol	Day 1	Day 2	Day 3	Day 4	Day 7	Day 14	Day 21

Control	1404 ± 96^a^	1401 ± 73^a^	1372 ± 56^a^	1691 ± 51^ab^	1671 ± 90^a^	1764 ± 223^a^	1273 ± 125^a^
Control + Hg	1450 ± 93^a^	1407 ± 38^a^	1396 ± 45^a^	1635 ± 52^a^	1575 ± 107^a^	1655 ± 89^a^	1274 ± 283^a^
Digestate	3558 ± 121^b^	2593 ± 574^b^	2733 ± 492^b^	4086 ± 116^d^	3776 ± 264^c^	3294 ± 380^b^	3757 ± 428^ab^
Digestate + Hg	3455 ± 569^b^	3190 ± 282^b^	3139 ± 328^b^	3804 ± 613^d^	4462 ± 644^c^	3975 ± 206^c^	4758 ± 327^b^
Ash	2335 ± 433^a^	2382 ± 197^a^	2281 ± 321^a^	2540 ± 234^bc^	2917 ± 164^b^	2686 ± 281^b^	2121 ± 286a^b^
Ash + Hg	1981 ± 254^a^	1908 ± 149^a^	2044 ± 161^a^	2574 ± 512^c^	2717 ± 117^b^	3092 ± 190^b^	2784 ± 467^ab^
(NH_4_)_2_SO_4_	1514 ± 57^a^	1356 ± 36^a^	1411 ± 102^a^	1651 ± 69^a^	1597 ± 79^a^	1684 ± 135^a^	1663 ± 92^ab^
(NH_4_)_2_SO_4_ + Hg	1655 ± 54^a^	1577 ± 246^a^	1431 ± 59^a^	1742 ± 108^abc^	1616 ± 32^a^	1666 ± 124^a^	1690 ± 107^ab^

**Table 4:** The concentrations of Mg extractable with 0.11 mol L^−1^ acetic acid within the incubation experiment (mg*·*kg^−1^); the averages marked by the same letter did not significantly differ at *P* < 0.05 within individual columns; data are presented as mean ± standard deviation (*n* = 3).

Chernozem	Day 1	Day 2	Day 3	Day 4	Day 7	Day 14	Day 21
Control	389 ± 20^a^	401 ± 2^ab^	483 ± 8^a^	562 ± 18^a^	503 ± 48^a^	546 ± 23^a^	371 ± 50^a^
Control + Hg	347 ± 111^a^	406 ± 20^abc^	442 ± 32^a^	556 ± 16^a^	502 ± 69^a^	505 ± 34^a^	533 ± 135^ab^
Digestate	544 ± 50^c^	544 ± 30^d^	603 ± 65^b^	753 ± 52^bc^	656 ± 76^ab^	781 ± 54^c^	963 ± 155^c^
Digestate + Hg	519 ± 37^c^	603 ± 24^e^	610 ± 45^b^	809 ± 44^d^	734 ± 73^b^	742 ± 22^bc^	859 ± 77^bc^
Ash	442 ± 24^ab^	458 ± 27^c^	522 ± 22^ab^	612 ± 22^ac^	566 ± 56^a^	574 ± 66^a^	636 ± 147^abc^
Ash + Hg	432 ± 57^ab^	447 ± 9^bc^	518 ± 35^ab^	681 ± 72^ab^	555 ± 31^a^	626 ± 62^ab^	585 ± 122^ab^
(NH_4_)_2_SO_4_	374 ± 3^a^	394 ± 16^ab^	461 ± 13^a^	572 ± 72^a^	506 ± 59^a^	494 ± 71^a^	626 ± 171^abc^
(NH_4_)_2_SO_4_ + Hg	406 ± 30^ab^	390 ± 10^a^	468 ± 32^a^	566 ± 10^a^	505 ± 45^a^	553 ± 39^a^	489 ± 119^a^

Luvisol	Day 1	Day 2	Day 3	Day 4	Day 7	Day 14	Day 21

Control	65 ± 5^a^	72 ± 5^a^	71 ± 2^a^	89 ± 3^a^	120 ± 47^a^	129 ± 61^a^	89 ± 19^a^
Control + Hg	70 ± 4^a^	72 ± 2^a^	73 ± 1^a^	86 ± 3^a^	108 ± 43^a^	115 ± 45^a^	82 ± 11^a^
Digestate	362 ± 27^c^	259 ± 70^c^	325 ± 52^b^	446 ± 5^c^	408 ± 40^b^	391 ± 51^b^	423 ± 11^c^
Digestate + Hg	364 ± 89^c^	336 ± 38^c^	363 ± 92^b^	460 ± 54^c^	476 ± 28^b^	416 ± 33^b^	366 ± 90^c^
Ash	144 ± 32^b^	156 ± 19^b^	162 ± 34^ab^	176 ± 21^b^	231 ± 51^a^	214 ± 66^a^	174 ± 40^ab^
Ash + Hg	116 ± 19^b^	121 ± 14^ab^	140 ± 21^ab^	178 ± 47^b^	214 ± 52^a^	243 ± 31^a^	240 ± 33^b^
(NH_4_)_2_SO_4_	73 ± 0^a^	68 ± 0^a^	72 ± 5^a^	85 ± 1^a^	120 ± 49^a^	114 ± 43^a^	120 ± 33^a^
(NH_4_)_2_SO_4_ + Hg	85 ± 9^a^	74 ± 5^ab^	76 ± 2^ab^	92 ± 7^a^	115 ± 46^a^	119 ± 50^a^	117 ± 38^a^

**Table 5:** The concentrations of K extractable with 0.11 mol L^−1^ acetic acid within the incubation experiment (mg*·*kg^−1^); the averages marked by the same letter did not significantly differ at *P* < 0.05 within individual columns; data are presented as mean ± standard deviation (*n* = 3).

Chernozem	Day 1	Day 2	Day 3	Day 4	Day 7	Day 14	Day 21
Control	68 ± 5^a^	78 ± 4^a^	78 ± 2^a^	84 ± 2^a^	92 ± 4^a^	95 ± 0^a^	53 ± 3^a^
Control + Hg	83 ± 17^a^	76 ± 4^a^	77 ± 10^a^	87 ± 3^a^	90 ± 6^a^	94 ± 3^a^	76 ± 12^a^
Digestate	2882 ± 906^b^	2931 ± 491^b^	3026 ± 304^b^	3547 ± 163^b^	3615 ± 177^c^	3842 ± 277^c^	4408 ± 506^b^
Digestate + Hg	2461 ± 510^b^	3163 ± 128^b^	2961 ± 261^b^	3915 ± 370^b^	3780 ± 164^d^	3697 ± 191^c^	4283 ± 367^b^
Ash	269 ± 66^a^	284 ± 40^a^	294 ± 59^a^	317 ± 78^a^	374 ± 35^cd^	362 ± 18^ab^	406 ± 146^a^
Ash + Hg	260 ± 61^a^	305 ± 77^a^	310 ± 47^a^	467 ± 101^a^	362 ± 25^bc^	446 ± 63^b^	359 ± 82^a^
(NH_4_)_2_SO_4_	97 ± 5^a^	119 ± 11^a^	107 ± 6^a^	130 ± 13^a^	126 ± 6^ab^	117 ± 13^ab^	109 ± 6^a^
(NH_4_)_2_SO_4_ + Hg	105 ± 6^a^	109 ± 4^a^	112 ± 6^a^	135 ± 2^a^	130 ± 5^abc^	145 ± 10^ab^	1568 ± 207^a^

Luvisol	Day 1	Day 2	Day 3	Day 4	Day 7	Day 14	Day 21

Control	117 ± 7^a^	126 ± 7^a^	117 ± 3^a^	140 ± 4^a^	138 ± 2^a^	147 ± 9^a^	111 ± 12^a^
Control + Hg	122 ± 6^a^	130 ± 6^a^	125 ± 4^a^	139 ± 4^a^	138 ± 5^a^	146 ± 3^a^	122 ± 21^a^
Digestate	3324 ± 201^b^	2684 ± 425^b^	3173 ± 54^b^	3857 ± 252^b^	3936 ± 37^c^	3665 ± 83^c^	4063 ± 418^c^
Digestate + Hg	3728 ± 541^b^	3306 ± 214^b^	3351 ± 540^b^	4065 ± 150^b^	3927 ± 308^c^	3655 ± 64^c^	3304 ± 180^b^
Ash	431 ± 146^a^	487 ± 78^a^	431 ± 111^a^	419 ± 70^a^	548 ± 66^b^	446 ± 71^b^	387 ± 18^a^
Ash + Hg	316 ± 70^a^	343 ± 41^a^	370 ± 39^a^	454 ± 102^a^	489 ± 15^b^	546 ± 66^b^	553 ± 118^a^
(NH_4_)_2_SO_4_	152 ± 3^a^	145 ± 5^a^	143 ± 11^a^	162 ± 1^a^	161 ± 5^a^	167 ± 9^a^	166 ± 17^a^
(NH_4_)_2_SO_4_ + Hg	159 ± 6^a^	158 ± 9^a^	152 ± 4^a^	176 ± 9^a^	169 ± 4^a^	162 ± 4^a^	170 ± 14^a^

**Table 6:** The concentrations of Cu extractable with 0.11 mol L^−1^ acetic acid within the incubation experiment (mg*·*kg^−1^); the averages marked by the same letter did not significantly differ at *P* < 0.05 within individual columns; data are presented as mean ± standard deviation (*n* = 3).

Chernozem	Day 1	Day 2	Day 3	Day 4	Day 7	Day 14	Day 21
Control	0.045 ± 0.007^a^	0.039 ± 0.008^a^	0.015 ± 0.003^a^	0.025 ± 0.003^a^	0.162 ± 0.018^abc^	0.064 ± 0.013^a^	0.017 ± 0.008^a^
Control + Hg	0.047 ± 0.012^a^	0.017 ± 0.001^a^	0.017 ± 0.004^a^	0.023 ± 0.003^a^	0.039 ± 0.019^a^	0.098 ± 0.025^a^	0.034 ± 0.015^a^
Digestate	0.224 ± 0.092^b^	0.230 ± 0.098^b^	0.187 ± 0.037^c^	0.299 ± 0.010^c^	0.362 ± 0.051^c^	0.517 ± 0.089^c^	0.911 ± 0.141^b^
Digestate + Hg	0.120 ± 0.010^a^	0.094 ± 0.026^a^	0.089 ± 0.028^b^	0.166 ± 0.038^b^	0.264 ± 0.031^bc^	0.334 ± 0.031^b^	0.763 ± 0.065^b^
Ash	0.053 ± 0.019^a^	0.059 ± 0.014^a^	0.032 ± 0.007^a^	0.054 ± 0.012^a^	0.065 ± 0.042^ab^	0.073 ± 0.043^a^	0.148 ± 0.029^a^
Ash + Hg	0.079 ± 0.011^a^	0.042 ± 0.003^a^	0.026 ± 0.005^a^	0.060 ± 0.013^a^	0.055 ± 0.035^a^	0.095 ± 0.027^a^	0.141 ± 0.069^a^
(NH_4_)_2_SO_4_	0.047 ± 0.035^a^	0.048 ± 0.013^a^	0.018 ± 0.006^a^	0.022 ± 0.006^a^	0.081 ± 0.031^ab^	0.091 ± 0.038^a^	0.090 ± 0.060^a^
(NH_4_)_2_SO_4_ + Hg	0.038 ± 0.020^a^	0.029 ± 0.007^a^	0.008 ± 0.008^a^	0.029 ± 0.002^a^	0.029 ± 0.013^a^	0.066 ± 0.048^a^	0.379 ± 0.093^ab^

Luvisol	Day 1	Day 2	Day 3	Day 4	Day 7	Day 14	Day 21

Control	0.095 ± 0.003^a^	0.084 ± 0.019^a^	0.061 ± 0.007^a^	0.134 ± 0.021^a^	0.142 ± 0.042^a^	0.226 ± 0.008^a^	0.252 ± 0.055^a^
Control + Hg	0.128 ± 0.020^a^	0.118 ± 0.021^ab^	0.098 ± 0.011^a^	0.156 ± 0.024^ab^	0.164 ± 0.062^a^	0.357 ± 0.021^ab^	0.230 ± 0.062^a^
Digestate	0.284 ± 0.085^a^	0.268 ± 0.022^b^	0.316 ± 0.051^b^	0.553 ± 0.087^c^	0.534 ± 0.136^b^	0.785 ± 0.032^d^	1.200 ± 0.142^c^
Digestate + Hg	0.166 ± 0.122^a^	0.095 ± 0.012^a^	0.120 ± 0.055^a^	0.131 ± 0.092^a^	0.119 ± 0.015^a^	0.594 ± 0.137^cd^	0.678 ± 0.164^ab^
Ash	0.196 ± 0.026^a^	0.161 ± 0.036^ab^	0.145 ± 0.023^ab^	0.269 ± 0.051^ab^	0.220 ± 0.045^a^	0.542 ± 0.046^bc^	0.493 ± 0.081^ab^
Ash + Hg	0.225 ± 0.048^ab^	0.261 ± 0.024^b^	0.193 ± 0.016^ab^	0.298 ± 0.045^b^	0.266 ± 0.040^a^	0.578 ± 0.058^c^	0.900 ± 0.042^bc^
(NH_4_)_2_SO_4_	0.101 ± 0.022^a^	0.191 ± 0.048^ab^	0.078 ± 0.004^a^	0.184 ± 0.018^ab^	0.161 ± 0.024^a^	0.308 ± 0.051^a^	0.658 ± 0.103^ab^
(NH_4_)_2_SO_4_ + Hg	0.140 ± 0.027^a^	0.138 ± 0.028^ab^	0.121 ± 0.014^a^	0.247 ± 0.068^ab^	0.193 ± 0.050^a^	0.422 ± 0.101^abc^	0.555 ± 0.062^ab^

**Table 7:** The concentrations of Fe extractable with 0.11 mol L^−1^ acetic acidwithin the incubation experiment (mg*·*kg^−1^); The averages marked by the same letter did not significantly differ at *P* < 0.05 within individual columns; data are presented as mean ± standard deviation (*n* = 3).

Chernozem	Day 1	Day 2	Day 3	Day 4	Day 7	Day 14	Day 21
Control	1.54 ± 0.38^a^	20.47 ± 6.26^a^	8.49 ± 0.70^a^	0.28 ± 0.02^a^	0.48 ± 0.11^a^	4.52 ± 0.24^a^	0.25 ± 0.06^a^
Control + Hg	1.40 ± 0.09^a^	8.20 ± 2.34^a^	10.76 ± 8.02^a^	0.18 ± 0.03^a^	0.20 ± 0.03^a^	2.90 ± 1.05^a^	0.24 ± 0.04^a^
Digestate	49.3 ± 9.4^c^	95.4 ± 17.2^c^	105.9 ± 2.6^b^	142.5 ± 11.7^c^	102.4 ± 2.7^c^	32.4 ± 5.3^b^	48.7 ± 6.4^b^
Digestate + Hg	27.0 ± 4.2^b^	61.9 ± 15.2^b^	77.1 ± 28.8^b^	73.1 ± 10.7^b^	69.4 ± 5.5^b^	48.0 ± 14.2^c^	48.3 ± 9.6^b^
Ash	1.31 ± 0.16^a^	28.78 ± 5.86^a^	6.87 ± 1.97^a^	0.26 ± 0.10^a^	0.35 ± 0.11^a^	1.53 ± 0.17^a^	0.90 ± 0.24^a^
Ash + Hg	1.16 ± 0.46^a^	8.17 ± 2.01^a^	7.58 ± 4.58^a^	0.30 ± 0.11^a^	0.28 ± 0.04^a^	1.07 ± 0.10^a^	0.06 ± 0.03^a^
(NH_4_)_2_SO_4_	1.29 ± 0.15^a^	17.20 ± 5.83^a^	5.53 ± 1.19^a^	0.35 ± 0.17^a^	0.26 ± 0.06^a^	3.91 ± 1.32^a^	0.15 ± 0.12^a^
(NH_4_)_2_SO_4_ + Hg	1.13 ± 0.28^a^	10.53 ± 3.15^a^	7.21 ± 2.97^a^	0.14 ± 0.09^a^	0.23 ± 0.03^a^	1.53 ± 0.19^a^	2.37 ± 0.36^a^

Luvisol	Day 1	Day 2	Day 3	Day 4	Day 7	Day 14	Day 21

Control	3.88 ± 2.13^a^	20.08 ± 6.85^a^	7.35 ± 1.27^a^	1.31 ± 0.18^a^	1.04 ± 0.14^a^	7.17 ± 2.64^a^	1.13 ± 0.66^a^
Control + Hg	2.21 ± 0.27^a^	10.02 ± 4.27^a^	12.24 ± 2.19^a^	1.38 ± 0.31^a^	0.91 ± 0.08^a^	5.99 ± 0.76^a^	0.67 ± 0.47^a^
Digestate	100.8 ± 33.1^b^	51.9 ± 25.4^b^	67.6 ± 40.6^ab^	44.7 ± 14.3^b^	77.4 ± 14.5^b^	42.5 ± 13.0^a^	173.5 ± 71.0^b^
Digestate + Hg	108.0 ± 38.7^b^	340.9 ± 82.3^b^	329.4 ± 70.4^b^	413.7 ± 52.8^c^	484.7 ± 97.2^c^	396.9 ± 54.1^b^	315.4 ± 63.8^c^
Ash	2.80 ± 0.70^a^	16.71 ± 3.74^a^	15.41 ± 3.44^a^	1.82 ± 0.59^a^	1.58 ± 0.77^a^	7.44 ± 2.02^a^	2.86 ± 1.43^a^
Ash + Hg	2.56 ± 0.27^a^	15.88 ± 9.53^a^	11.24 ± 4.10^a^	1.21 ± 0.44^a^	0.99 ± 0.18^a^	2.94 ± 1.18^a^	0.59 ± 0.05^a^
(NH_4_)_2_SO_4_	3.07 ± 0.09^a^	8.82 ± 0.53^a^	7.06 ± 2.55^a^	1.61 ± 0.21^a^	1.28 ± 0.07^a^	5.68 ± 1.15^a^	1.64 ± 0.30^a^
(NH_4_)_2_SO_4_ + Hg	3.01 ± 0.32^a^	10.36 ± 2.32^a^	14.56 ± 2.55^a^	1.59 ± 0.21^a^	1.39 ± 0.24^a^	2.47 ± 0.42^a^	1.02 ± 0.23^a^

**Table 8:** The concentrations of Mn extractable with 0.11 mol L^−1^ acetic acid within the incubation experiment (mg*·*kg^−1^); the averages marked by the same letter did not significantly differ at *P* < 0.05 within individual columns; data are presented as mean ± standard deviation (*n* = 3).

Chernozem	Day 1	Day 2	Day 3	Day 4	Day 7	Day 14	Day 21
Control	19.5 ± 1.2^a^	19.9 ± 0.7^a^	22.2 ± 1.1^a^	35.0 ± 6.1^a^	33.9 ± 0.9^a^	46.7 ± 16.0^a^	34.7 ± 8.7^a^
Control + Hg	21.4 ± 4.4^a^	22.1 ± 3.4^ab^	20.7 ± 3.9^a^	36.2 ± 4.8^a^	36.6 ± 0.3^a^	37.3 ± 5.0^c^	37.8 ± 11.6^a^
Digestate	168 ± 22^c^	168 ± 12^c^	177 ± 10^c^	232 ± 4^d^	204 ± 4^c^	218 ± 11^a^	301 ± 68^d^
Digestate + Hg	152 ± 4^c^	181 ± 6^c^	168 ± 4^c^	229 ± 23^d^	204 ± 5^c^	208 ± 3^c^	266 ± 30^cd^
Ash	42.4 ± 7.3^b^	38.7 ± 9.8^ab^	57.5 ± 21.7^b^	70.2 ± 8.5^bc^	66.3 ± 10.6^b^	66.9 ± 14.0^a^	101.5 ± 53.7^ab^
Ash + Hg	38.5 ± 9.2^b^	40.0 ± 4.4^b^	46.1 ± 7.0a^b^	96.8 ± 18.0^c^	73.9 ± 6.5^b^	93.1 ± 15.5^ab^	83.0 ± 32.3^ab^
(NH_4_)_2_SO_4_	23.8 ± 1.5^a^	24.1 ± 1.6^ab^	27.0 ± 0.7^a^	46.7 ± 5.0^ab^	69.7 ± 16.6^b^	143.1 ± 19.7^b^	125.6 ± 36.1^bc^
(NH_4_)_2_SO_4_ + Hg	26.4 ± 8.5^a^	28.0 ± 7.4^ab^	30.3 ± 9.3^ab^	39.2 ± 5.5^ab^	40.7 ± 9.8^a^	56.5 ± 13.9^a^	70.8 ± 33.6^ab^

Luvisol	Day 1	Day 2	Day 3	Day 4	Day 7	Day 14	Day 21

Control	27.0 ± 1.0^a^	30.3 ± 1.0^a^	33.6 ± 3.6^a^	55.9 ± 1.0^a^	50.8 ± 9.3^a^	64.2 ± 14.0^a^	34.7 ± 7.8^a^
Control + Hg	43.6 ± 0.8^abc^	43.3 ± 10.1^ab^	45.1 ± 7.0^abc^	67.4 ± 5.1^ab^	61.5 ± 6.7^a^	77.4 ± 8.5^ab^	37.8 ± 7.1^a^
Digestate	161 ± 9^d^	142 ± 23^d^	169 ± 8^d^	191 ± 2^c^	198 ± 25^c^	189 ± 19^c^	301 ± 30^c^
Digestate + Hg	148 ± 9^d^	176 ± 2^e^	180 ± 5^d^	222 ± 10^d^	223 ± 11^c^	229 ± 11^d^	266 ± 81^c^
Ash	54.9 ± 10.4^c^	59.4 ± 8.8^b^	53.2 ± 9.0^bc^	87.0 ± 14.2^b^	131.4 ± 42.3^b^	114.0 ± 23.8^b^	101.5 ± 34.1^b^
Ash + Hg	54.4 ± 7.2^c^	58.6 ± 2.4^b^	58.9 ± 4.8^c^	86.0 ± 12.1^b^	81.9 ± 5.8^ab^	108.9 ± 0.8^b^	83.0 ± 69.1^a^
(NH_4_)_2_SO_4_	27.9 ± 0.5^ab^	28.5 ± 1.5^a^	29.0 ± 1.7^a^	50.3 ± 8.3^a^	42.8 ± 6.6^a^	60.9 ± 12.4^a^	125.6 ± 3.2^b^
(NH_4_)_2_SO_4_ + Hg	46.5 ± 4.6^bc^	46.9 ± 5.2^ab^	42.6 ± 3.2^ab^	62.4 ± 12.4^ab^	65.9 ± 6.0^a^	63.6 ± 2.9^a^	70.8 ± 5.2^a^

**Table 9:** The concentrations of P extractable with 0.11 mol L^−1^ acetic acid within the incubation experiment (mg*·*kg^−1^); the averages marked by the same letter did not significantly differ at *P* < 0.05 within individual columns; data are presented as mean ± standard deviation (*n* = 3).

Chernozem	Day 1	Day 2	Day 3	Day 4	Day 7	Day 14	Day 21
Control	90.9 ± 6.3^a^	88.9 ± 24.9^a^	112.3 ± 33.3^a^	109.3 ± 7.0^a^	93.6 ± 7.9^a^	88.8 ± 11.7^a^	55.9 ± 6.5^a^
Control + Hg	85.0 ± 17.9^a^	88.1 ± 1.9^a^	108.4 ± 19.0^a^	127.8 ± 14.3^a^	91.2 ± 4.7^a^	98.1 ± 14.1^a^	79.3 ± 13.9^a^
Digestate	259.0 ± 50.0^b^	269.0 ± 49.5^b^	235.2 ± 63.2^b^	320.9 ± 60.5^b^	189.1 ± 23.5^b^	278.6 ± 30.5^b^	246.0 ± 59.9^b^
Digestate + Hg	250.9 ± 62.4^b^	285.6 ± 28.3^b^	261.6 ± 40.4^b^	361.0 ± 14.2^b^	293.6 ± 12.5^c^	274.5 ± 51.7^b^	253.5 ± 51.8^b^
Ash	107.1 ± 6.2^a^	102.1 ± 17.1^a^	101.7 ± 2.9^a^	130.0 ± 18.1^a^	115.4 ± 17.5^a^	98.9 ± 9.2^a^	123.3 ± 30.4^a^
Ash + Hg	104.2 ± 7.5^a^	101.6 ± 5.7^a^	105.5 ± 6.4^a^	138.3 ± 11.9^a^	118.4 ± 28.0^a^	115.3 ± 6.0^a^	96.6 ± 19.6^a^
(NH_4_)_2_SO_4_	96.9 ± 10.0^a^	91.3 ± 1.7^a^	93.4 ± 13.1^a^	110.4 ± 4.8^a^	94.4 ± 1.2^a^	102.2 ± 12.6^a^	116.8 ± 15.6^a^
(NH_4_)_2_SO_4_ + Hg	84.3 ± 4.7^a^	90.2 ± 4.3^a^	117.9 ± 22.3^a^	118.7 ± 17.1^a^	95.4 ± 3.1^a^	91.8 ± 7.9^a^	106.5 ± 37.4^a^

Luvisol	Day 1	Day 2	Day 3	Day 4	Day 7	Day 14	Day 21

Control	62.9 ± 5.8^a^	61.9 ± 6.3^a^	61.2 ± 11.1^a^	70.8 ± 0.6^a^	65.8 ± 16.3^a^	56.62.7 ± ^a^	38.9 ± 3.8^a^
Control + Hg	66.8 ± 9.6^a^	63.4 ± 0.5^a^	65.6 ± 10.4^a^	71.5 ± 8.6^a^	62.3 ± 1.7^a^	67.4 ± 5.7^a^	43.7 ± 12.0^a^
Digestate	323.7 ± 26.2^b^	210.2 ± 67.6^b^	203.4 ± 58.7^b^	323.4 ± 14.3^b^	290.3 ± 30.1^b^	245.8 ± 56.8^b^	254.0 ± 24.0^c^
Digestate + Hg	329.6 ± 82.9^b^	286.5 ± 40.6^b^	246.3 ± 77.2^b^	350.0 ± 44.8^b^	348.6 ± 90.4^b^	262.2 ± 28.7^b^	130.7 ± 48.9^b^
Ash	85.0 ± 5.3^a^	84.4 ± 8.8^a^	80.5 ± 13.1^a^	90.6 ± 14.1^a^	92.6 ± 7.4^a^	78.3 ± 4.8^a^	55.6 ± 6.0^a^
Ash + Hg	76.4 ± 6.3^a^	73.7 ± 4.7^a^	75.8 ± 3.6^a^	90.7 ± 12.4^a^	81.4 ± 2.5^a^	86.5 ± 4.0^a^	68.7 ± 1.6^a^
(NH_4_)_2_SO_4_	61.3 ± 1.1^a^	58.3 ± 3.6^a^	57.3 ± 4.0^a^	69.1 ± 8.2^a^	59.4 ± 4.8^a^	59.3 ± 4.2^a^	60.6 ± 9.2 ^a^
(NH_4_)_2_SO_4_ + Hg	60.4 ± 1.6^a^	72.9 ± 13.7^a^	59.6 ± 2.8^a^	69.5 ± 1.2^a^	63.3 ± 4.1^a^	58.8 ± 2.5^a^	58.7 ± 0.6^a^

**Table 10:** The concentrations of S extractable with 0.11 mol L^−1^ acetic acid within the incubation experiment (mg*·*kg^−1^); the averages marked by the same letter did not significantly differ at *P* < 0.05 within individual columns; data are presented as mean ± standard deviation (*n* = 3).

Chernozem	Day 1	Day 2	Day 3	Day 4	Day 7	Day 14	Day 21
Control	8.8 ± 1.0^a^	9.7 ± 0.9^a^	12.3 ± 5.6^a^	14.9 ± 5.0^a^	17.7 ± 2.1^a^	16.1 ± 4.1^a^	10.0 ± 2.7^a^
Control + Hg	9.3 ± 0.6^a^	9.6 ± 0.6^a^	9.6 ± 1.5^a^	13.0 ± 2.4^a^	15.1 ± 4.3^a^	15.2 ± 3.7^a^	15.5 ± 4.6^a^
Digestate	255.0 ± 114.1^bc^	199.5 ± 54.8^b^	129.0 ± 31.7^b^	89.7 ± 21.3^ab^	66.1 ± 3.5^ab^	132.2 ± 52.9^ab^	211.3 ± 93.9^bc^
Digestate + Hg	141.4 ± 30.4^b^	221.6 ± 29.6^b^	162.7 ± 46.2^b^	198.1 ± 63.6^ab^	91.6 ± 6.7^ab^	136.4 ± 49.1^ab^	307.6 ± 110.8^cd^
Ash	138.1 ± 37.6^b^	140.8 ± 32.9^b^	143.9 ± 33.0^b^	153.8 ± 37.3^ab^	168.1 ± 12.0^b^	165.4 ± 16.1^ab^	201.9 ± 62.7^bc^
Ash + Hg	138.2 ± 20.2^b^	158.9 ± 7.5^b^	155.2 ± 21.9^b^	243.6 ± 41.8^ab^	192.6 ± 3.3^b^	205.0 ± 28.7^b^	202.5 ± 54.7^bc^
(NH_4_)_2_SO_4_	344.0 ± 82.0^c^	494.3 ± 153.6^c^	454.5 ± 79.6^c^	602.6 ± 145.5^c^	483.6 ± 55.2^c^	528.4 ± 91.7^c^	519.6 ± 104.6^e^
(NH_4_)_2_SO_4_ + Hg	366.3 ± 66.7^c^	445.8 ± 94.6^c^	386.7 ± 13.1^c^	660.6 ± 189.3^c^	483.2 ± 162.3^c^	766.5 ± 78.7^d^	429.7 ± 123.6^de^

Luvisol	Day 1	Day 2	Day 3	Day 4	Day 7	Day 14	Day 21

Control	4.6 ± 1.0^a^	4.9 ± 1.0^a^	5.3 ± 1.1^a^	8.6 ± 2.3^a^	11.1 ± 3.6^a^	11.8 ± 6.3^a^	9.2 ± 3.3^a^
Control + Hg	5.0 ± 1.0^a^	4.7 ± 0.3^a^	5.7 ± 0.4^a^	9.8 ± 6.1^a^	11.4 ± 5.5^a^	10.2 ± 4.9^a^	7.0 ± 0.6^a^
Digestate	296.4 ± 27.4^bc^	221.6 ± 27.5^bc^	275.5 ± 32.2^bc^	420.5 ± 122.4^cd^	329.1 ± 62.0^cd^	220.3 ± 121.1^b^	264.9 ± 101.4^b^
Digestate + Hg	273.2 ± 55.6^bc^	205.9 ± 19.7^bc^	234.2 ± 46.2^bc^	229.7 ± 48.9^bc^	80.3 ± 26.6^ab^	94.9 ± 11.1a^b^	362.5 ± 93.2^b^
Ash	183.2 ± 75.2^b^	183.2 ± 20.0^b^	214.5 ± 64.0^b^	208.2 ± 15.6^b^	223.6 ± 13.8^bc^	198.3 ± 14.0^b^	151.0 ± 27.1^b^
Ash + Hg	134.5 ± 21.6^b^	131.6 ± 11.8^b^	152.8 ± 21.2^b^	219.1 ± 27.2^bc^	233.4 ± 34.5^bc^	228.7 ± 40.6^b^	203.4 ± 36.8^b^
(NH_4_)_2_SO_4_	443.5 ± 96.3^c^	403.0 ± 96.0^c^	466.6 ± 87.8^c^	590.4 ± 130.9^d^	430.3 ± 97.5^d^	505.2 ± 108.6^c^	570.8 ± 118.5^c^
(NH_4_)_2_SO_4_ + Hg	536.1 ± 61.6^c^	564.1 ± 30.2^c^	541.0 ± 78.0^c^	687.8 ± 87.9^e^	560.1 ± 24.8^e^	564.4 ± 72.6^c^	701.8 ± 31.8^c^

**Table 11:** The concentrations of Zn extractable with 0.11 mol L^−1^ acetic acid within the incubation experiment (mg*·*kg^−1^); the averages marked by the same letter did not significantly differ at *P* < 0.05 within individual columns; data are presented as mean ± standard deviation (*n* = 3).

Chernozem	Day 1	Day 2	Day 3	Day 4	Day 7	Day 14	Day 21
Control	1.34 ± 0.07^a^	1.37 ± 0.08^a^	0.87 ± 0.03^a^	1.57 ± 0.19^a^	1.42 ± 0.40^a^	1.32 ± 0.26^a^	0.59 ± 0.02^a^
Control + Hg	1.43 ± 0.29^a^	1.27 ± 0.06^a^	0.88 ± 0.15^a^	1.36 ± 0.19^a^	1.28 ± 0.08^a^	1.28 ± 0.16^a^	0.97 ± 0.33^a^
Digestate	6.56 ± 0.81^a^	6.69 ± 1.21^a^	6.02 ± 1.11^a^	9.02 ± 0.93^a^	7.44 ± 0.26^a^	11.06 ± 1.22^a^	12.51 ± 1.37^a^
Digestate + Hg	5.23 ± 1.36^a^	5.74 ± 0.82^a^	5.21 ± 0.84^a^	7.76 ± 0.34^a^	6.69 ± 0.26^a^	7.89 ± 0.17^a^	9.98 ± 1.51^a^
Ash	109.3 ± 23.2^b^	52.1 ± 20.0^b^	85.8 ± 21.8^b^	93.9 ± 23.7^b^	102.9 ± 12.2^b^	77.2 ± 17.3^b^	126.6 ± 32.9^b^
Ash + Hg	52.6 ± 25.8^b^	47.1 ± 10.4^b^	81.5 ± 21.4^b^	153.8 ± 25.7^c^	108.5 ± 31.6^b^	137.6 ± 22.4^c^	154.3 ± 28.8^b^
(NH_4_)_2_SO_4_	1.57 ± 0.29^a^	1.55 ± 0.15^a^	1.12 ± 0.09^a^	1.67 ± 0.07^a^	1.66 ± 0.20^a^	2.29 ± 0.27^a^	2.27 ± 0.32^a^
(NH_4_)_2_SO_4_ + Hg	1.65 ± 0.26^a^	1.39 ± 0.08^a^	1.15 ± 0.18^a^	1.44 ± 0.13^a^	1.45 ± 0.13^a^	1.56 ± 0.06^a^	5.02 ± 0.35^a^

Luvisol	Day 1	Day 2	Day 3	Day 4	Day 7	Day 14	Day 21

Control	2.66 ± 0.10^a^	2.92 ± 0.19^a^	2.46 ± 0.21^a^	3.45 ± 0.19^a^	3.21 ± 0.12^a^	3.15 ± 0.10^a^	2.02 ± 0.39^a^
Control + Hg	2.94 ± 0.06^a^	2.99 ± 0.30^a^	2.64 ± 0.15^a^	4.84 ± 2.17^a^	3.05 ± 0.04^a^	3.26 ± 0.06^a^	2.41 ± 0.89^a^
Digestate	11.58 ± 0.67^a^	8.13 ± 2.02^a^	8.53 ± 1.57^a^	14.04 ± 0.43^a^	13.07 ± 0.99^a^	12.49 ± 1.14^a^	14.68 ± 1.98^a^
Digestate + Hg	9.02 ± 1.29^a^	8.33 ± 1.04^a^	7.86 ± 0.69^a^	10.24 ± 0.52^a^	9.98 ± 0.73^a^	11.85 ± 1.90^a^	6.81 ± 4.38^a^
Ash	181.6 ± 30.7^b^	172.9 ± 28.8^b^	161.4 ± 32.8^b^	164.6 ± 31.0^b^	214.2 ± 24.5^c^	155.6 ± 25.8^b^	115.8 ± 23.0^b^
Ash + Hg	112.4 ± 31.8^b^	92.2 ± 12.3^b^	121.1 ± 23.3^b^	165.8 ± 34.6^b^	162.6 ± 4.6^b^	201.9 ± 35.7^b^	230.9 ± 38.7^c^
(NH_4_)_2_SO_4_	3.05 ± 0.18^a^	3.16 ± 0.73^a^	2.65 ± 0.04^a^	3.74 ± 0.21^a^	3.38 ± 0.13^a^	3.98 ± 0.50^a^	4.39 ± 1.42^a^
(NH_4_)_2_SO_4_ + Hg	3.37 ± 0.24^a^	3.17 ± 0.28^a^	2.95 ± 0.24^a^	4.18 ± 0.08^a^	3.18 ± 0.31^a^	3.44 ± 0.07^a^	3.46 ± 0.35^a^
